# Clinical Significance of Preoperative Albumin and Globulin Ratio in Patients with Gastric Cancer Undergoing Treatment

**DOI:** 10.1155/2017/3083267

**Published:** 2017-03-23

**Authors:** Min-jie Mao, Xiao-li Wei, Hui Sheng, Xue-ping Wang, Xiao-hui Li, Yi-jun Liu, Shan Xing, Qi Huang, Shu-qin Dai, Wan-li Liu

**Affiliations:** ^1^Department of Clinical Laboratory, Sun Yat-sen University Cancer Center, State Key Laboratory of Oncology in South China, Collaborative Innovation Center for Cancer Medicine, 651 Dong Feng Road East, Guangzhou, Guangdong Province 510060, China; ^2^Department of Medical Oncology, Sun Yat-sen University Cancer Center, State Key Laboratory of Oncology in South China, Collaborative Innovation Center for Cancer Medicine, 651 Dong Feng Road East, Guangzhou, Guangdong Province 510060, China; ^3^Department of Experimental Research, Sun Yat-sen University Cancer Center, State Key Laboratory of Oncology in South China, Collaborative Innovation Center for Cancer Medicine, 651 Dong Feng Road East, Guangzhou, Guangdong Province 510060, China; ^4^Guangdong Medical University, 1 Xin Cheng Road, Songshan Hu, Dongguan, Guangdong Province 523808, China

## Abstract

*Background*. The pretreatment albumin and globulin ratio (AGR) was an inflammation-associated factor which was related to the overall survival in various malignancies. The aim of this study was to evaluate the prognostic value of AGR in patients with gastric cancer.* Method*. This retrospective study included 862 cases pathologically diagnosed with gastric cancer. All patients were randomly divided into the testing group (431 cases) and validation group (431 cases). The relationships of AGR with clinicopathologic characteristics and prognosis were analyzed by Kaplan-Meier and Cox regression methods.* Results*. In the testing group, the median overall survival was 26.90 months and the cutoff value of AGR was 1.50 based on R language. Kaplan-Meier analysis showed that lower AGR was correlated with poorer overall survival. Multivariate analysis demonstrated that AGR was an independent prognostic factor for overall survival (HR: 0.584, 95% CI = 0.351–0.973, and* p =* 0.039). In the validation group, the median overall survival was 24.10 months. Lower AGR (≤1.50) also had a significantly poorer overall survival by Kaplan-Meier analysis. According to multivariate analysis, the AGR was also confirmed to be an independent prognostic factor for overall survival (HR: 0.578, 95% CI = 0.373–0.897, and* p* = 0.015).* Conclusions*. Our study suggested that the pretreatment AGR could be a prognostic biomarker for overall survival in patients with gastric cancer.

## 1. Introduction

Gastric cancer (GC) is a common malignant tumor of upper digestive tract. In 2012, the incidence and mortality of GC in the world ranked fifth and third, with approximately 951,000 new cases and 723,000 deaths, respectively [1]. The most common pathologic type is adenocarcinoma [2]. Many techniques, such as the serum CA72-4 level testing and gastroscopy, have been used to screen for GC and assess the risk of recurrence. However, these biomarkers are not sufficient to predict prognosis accurately [3]. The surgical treatment is still the preferred treatment for GC patients without distant metastasis [4]. Although there was great advance in the diagnosis and treatment strategy, the prognosis was still poor; the five-year overall survival was only 4% for patients at stage IV [5]. Currently, the best clinical prognostic indicator for GC is TNM category, but there were still significant differences in prognosis among patients at the same stage [6]. Thus, it is important to look for clinical indicators which were reliable and easily detectable for survival and treatment guidance in GC.

Tumor development and metastasis could be promoted by chronic inflammation [7]. The interaction between tumors and immune system promoted the production of proinflammatory factors, which induced the occurrence of inflammation [8]. Systemic inflammatory response could inhibit immune surveillance, enhance the permeability of blood vessels and lymphatics, and degrade extracellular matrix by cytokines, chemokines, prostaglandins, and active amine, causing tumor development and metastasis [8]. It had been confirmed by previous reports that some inflammatory factors had significant prognostic value in GC, such as NLR (neutrophil/lymphocyte ratio) [9], PLR (platelet/lymphocyte ratio) [10], and CRP (C-reaction protein). Albumin (ALB) and globulin (GLB) were the major protein components in serum, which played an important role in inflammation [11, 12]. A decreased ALB level and increased GLB level had been reported to reflect chronic inflammation. Previously, ALB/GLB ratio (AGR) was mainly used for the diagnosis of liver function and immunological diseases [13]. In recent years, it has been reported as a novel inflammatory indicator for prognosis in colorectal cancer [14], lung cancer [15], esophageal cancer [16], and breast cancer [17]. But the prognostic value of AGR in GC remained to be further investigated.

In this study, we would assess the prognostic value of the pretreatment AGR for overall survival (OS) and evaluate its association with clinicopathologic characteristics and other inflammation-associated factors in GC patients with a large sample.

## 2. Materials and Methods

### 2.1. Patients

We retrospectively reviewed the clinical data of 1161 GC patients treated at Sun Yat-sen University Cancer Center, from 2009 to 2013. The main clinical characteristics are described in Table 1. All patients were pathological diagnosed with GC and classified and staged based on the American Joint Committee on Cancer (AJCC) tumor-node-metastasis (TNM) staging system. Exclusion criteria were as follows: (1) patients treated with medication or taking nutrition replacement therapy or any drugs known to affect serum ALB and GLB levels before serum collection; (2) patients with concomitant diseases associated with increased serum ALB and GLB levels (i.e., liver diseases, or metabolic syndrome); (3) other types of malignancy; (4) patients who were lost to follow-up. The serum detection results before initial treatment were all retrospectively obtained from the clinical laboratory. The globulin was calculated by total proteins minus albumin. The AGR was calculated with the equation: albumin/(total proteins − albumin). The NLR and PLR were calculated as neutrophil/lymphocyte ratio and platelet/lymphocyte ratio, respectively. Other clinicopathologic characteristics were retrospectively collected by reviewing medical records. Finally, there were 862 cases included in the study.

### 2.2. Laboratory Measurements

Peripheral blood was collected from the patients between 7 and 8 a.m., clotted at room temperature, and centrifuged at 3500 r/min for 8 min. The concentrations of albumin and total proteins were measured using a Hitachi 7600 automatic biochemical analyzer (Hitachi High-Technologies, Tokyo, Japan). Albumin and total proteins were measured using colorimetry. All reagents used in this study were provided by Wako Pure Chemical Industries, Japan. The coefficient of variation of the two tests in our laboratory is ≤5%. The levels of neutrophil, lymphocyte, and platelet were derived from the blood count using a Sysmex XE-5000 automatic blood-cell counter (Sysmex Corporation, Kobe, Japan). All the markers were measured before treatment after diagnosis in hospital. Venous blood samples were collected in tubes without anticoagulation at 7 am in the second day in hospital. The samples were centrifuged half an hour later and tested within 2 h of processing according to the instructions from the manufacturer.

### 2.3. Follow-Up

All patients provided written informed consent for the information to be used in our hospital database. Study approval was obtained from an independent ethics committee at Sun Yat-sen University Cancer Center. This study was conducted in accordance with the ethical standards of the World Medical Association Declaration of Helsinki. In our institution, patients were generally followed up every 3 months in the first years, every 6 months for the following 2 years, and annually thereafter. The last follow-up was in June 2016, survival status was verified again through checking clinical attendance records and direct telecommunication with the patients or their family (performed by The Medical Information Unit in our Cancer Center), and all the dead patients died of gastric cancer.

### 2.4. Statistical Analysis

We divided the patients into the testing group and the validation group with the equal numbers by random assignment. The AGR was categorized using the method reported by Budczies et al. [18]; NLR and PLR were also expressed as the median. The significance of correlations between the pretreatment AGR and the clinicopathologic characteristics was analyzed by *χ*2 test or Kruskal-Wallis* H* test. The associations of AGR with NLR and PLR were examined by correlation analysis. OS was the time interval from diagnosis of GC to death or the last follow-up and was analyzed using the Kaplan-Meier method. The differences in OS were assessed with the log-rank test. Univariate and multivariate analyses of clinical variables were performed using Cox proportional hazards regression models. All statistical analyses were performed using the SPSS 17.0 (SPSS Inc., Chicago, IL, USA). A two-tailed *p* value of <0.05 was considered significant.

## 3. Results

### 3.1. Patients' Baseline Characteristics in GC Patients

In this study, a total of 1161 patients with GC were screened and 862 patients were finally included for analyses. We divided the patients into the testing group (431 cases) and the validation group (431 cases) by random assignment. The clinicopathologic characteristics in each group were presented in Table 1. In the testing group, there were 288 (66.8%) male patients and 143 (33.2%) female patients. The median age was 59 (range: 26–85) years old. The number of patients at stages I, II, III, and IV was 66 (15.3%), 110 (25.5%), 201 (46.6%), and 54 (12.5%), respectively. There were 218 (50.6%) patients with proximal GC, 160 (37.1%) patients with remote GC, and 53 (12.3%) patients with GC in other locations. In the validating group, there were 298 (69.1%) male and 133 (30.9%) female patients. The median age was 59 (range: 26–85) years old. The number of patients at stages I, II, III, and IV was 58 (13.5%), 100 (23.2%), 208 (48.3%), and 65 (15.1%), respectively. There were 239 (55.5%) patients with proximal GC, 179 (41.5%) patients with remote GC, and 45 (10.4%) patients with GC in other locations.

### 3.2. The Prognostic Value of AGR for OS in All the GC Patients

In univariate analysis, age (HR: 1.50, 95% CI = 1.073–1.698, and* p* = 0.010), primary tumor size (HR: 1.779, 95% CI = 1.335–2.370, and *p* < 0.001), tumor location (HR: 1.195, 95% CI = 1.007–1.419, and* p* = 0.042), distant metastasis (HR: 6.113, 95% CI = 4.787–7.807, and *p* < 0.001), surgery (HR: 0.469, 95% CI = 0.358–0.613, and *p* < 0.001), the TNM category (HR: 5.320, 95% CI = 3.246–8.721, and *p* < 0.001), ALB (HR: 0.695, 95% CI = 0.552–0.875, and* p* = 0.002), PLR (HR: 1.730, 95% CI = 1.367–2.190, and *p* < 0.001), NLR (HR: 1.94, 95% CI = 1.517–2.466, and *p* < 0.001), and AGR (HR: 0.648, 95% CI = 0.514–0.817, and *p* < 0.001) were significantly associated with OS. Multivariate analysis (Cox proportional hazards model) identified distant metastasis (HR: 2.247, 95% CI = 1.504–3.356, and *p* < 0.001), the TNM category (HR: 2.962, 95% CI = 1.627–5.393, and *p* < 0.001), NLR (HR: 1.442, 95% CI = 1.028–2.023, and* p* = 0.011), and AGR (HR: 0.630, 95% CI = 0.451–0.879, and* p* = 0.007) as independent prognostic factors. ALB and GLB were not significantly associated with OS (see Supplement 1 in Supplementary Material available online at https://doi.org/10.1155/2017/3083267).

### 3.3. The Prognostic Value of AGR for OS in the Testing Group

The median OS for patients in the testing group was 26.90 months, with a range of 0.20–53.87 months. The median OS for patients with a higher level (>1.50) and lower level (≤1.50) of AGR were 30.00 and 24.10 months, respectively. In univariate analysis, the following factors were significantly associated with OS: primary tumor size (HR: 1.855, 95% CI = 1.182–2.909, and* p =* 0.007), tumor location (HR: 1.366, 95% CI = 1.046–1.784, and* p =* 0.022), distant metastasis (HR: 6.989, 95% CI = 4.776–10.228, and *p* < 0.001), surgery (HR: 0.509, 95% CI = 0.337–0.768, and *p* < 0.001), the TNM category (HR: 4.577, 95% CI = 2.859–7.326, and *p* < 0.001), PLR (HR: 1.923, 95% CI = 1.330–2.778, and* p =* 0.001), NLR (HR: 1.959, 95% CI = 1.344–2.855, and *p* < 0.001), and AGR (HR: 0.626, 95% CI = 0.438–0.859, and* p =* 0.001). Multivariate analysis (Cox proportional hazards model) was carried out based on all the potentially prognostic factors identified in univariate analysis above, to evaluate whether these factors were independent prognostic factors for survival. The results identified distant metastasis (HR: 2.361, 95% CI = 1.216–4.585, and* p* = 0.011) and AGR (HR: 0.584, 95% CI = 0.351–0.973, and* p* = 0.039) as independent prognostic factors (Table 2).

**Figure 1:**
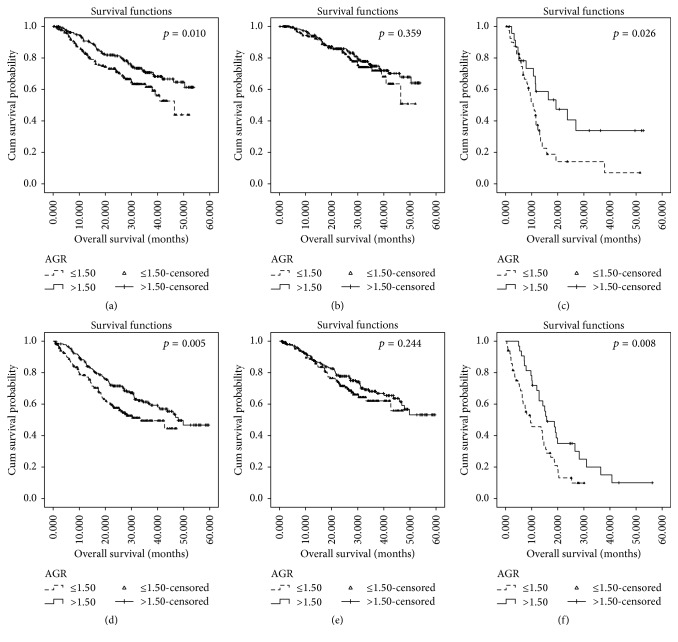
The prognostic significance of AGR based on the clinical stage in testing and validation group of GC. The five-year overall survival rate was calculated using the Kaplan-Meier method and analyzed with the log-rank test. A lower AGR level experienced significantly shorter overall survival than patients with a normal AGR in the whole cohort (a), stage I-II subgroup (b), and stage III-IV (c) of the testing group. Meanwhile, a lower AGR level experienced significantly shorter overall survival than patients with a normal AGR in the whole cohort (d), stage I-II subgroup (e), and stage III-IV (f) of the validation group in GC.

In the Kaplan-Meier analysis, AGR was significantly associated with OS (Figure 1(a)). For the whole cohort, the OS was 6.25 months shorter in patients with lower AGR (mean, 22.25 months) than those with higher AGR (mean, 28.50 months). As an important prognostic factor for OS, the relationship between AGR and survival was further evaluated according to the clinical stage. The survival rate of patients with decreased AGR levels had a significantly shorter OS compared with those patients with increased AGR levels only in advanced stage subgroup (*p* = 0.026, Figure 1(c)).

### 3.4. The Prognostic Value of AGR for OS in the Validation Group

The median OS for patients in the validation group was 24.10 months, with a range of 0.50–59.57 months. The median OS for patients with a higher level (>1.50) and a lower level (≤1.50) of AGR were 27.20 months and 20.00 months, respectively. In univariate analysis, age (HR: 1.015, 95% CI = 1.002–1.029, and* p* = 0.021), primary tumor size (HR: 1.713, 95% CI = 1.180–2.487, and* p =* 0.005), distant metastasis (HR: 5.375, 95% CI = 3.898–7.410, and* p* = 0.000), surgery (HR: 0.430, 95% CI = 0.307–0.626, and *p* < 0.001), the TNM category (HR: 6.205, 95% CI = 3.035–12.678, and *p* < 0.001), PLR (HR: 1.630, 95% CI = 1.199–2.215, and* p =* 0.002), NLR (HR: 1.946, 95% CI = 1.415–2.676, and *p* < 0.001), and AGR (HR: 0.649, 95% CI = 0.478–0.882, and* p =* 0.006) were significantly associated with OS. Multivariate analysis (Cox proportional hazards model) identified distant metastasis (HR: 2.939, 95% CI = 1.781–4.850, and *p* < 0.001), NLR (HR: 1.766, 95% CI = 1.136–2.745, and* p* = 0.011), and AGR (HR: 0.578, 95% CI = 0.373–0.897, and* p* = 0.015) as independent prognostic factors (Table 3).

In the Kaplan-Meier analysis, AGR was also closely associated with OS (Figure 1(d)). For the whole cohort, the OS was 7.90 months shorter in patients with lower AGR (mean, 19.73 months) than those with higher AGR (mean, 27.63 months), and the relationship between AGR and OS was further evaluated according to the TNM category. Patients with decreased AGR levels had a significantly shorter OS compared with those with increased AGR levels only in advanced stage subgroup (*p* = 0.008, Figure 1(f)).

## 4. Discussion

Recently, Chen et al. reported that a low level of GLB might be a significant prognostic marker for worse survival in patients with GC and they also mentioned the prognostic value of AGR in GC [19]. However, there were some limitations in their study. Firstly, they did not explore the relationship between AGR and clinical pathological characteristics of GC patients. Secondly, they only studied the prognostic value of AGR for patients who received radical surgery. For patients who did not receive surgical treatment, the prognostic role of AGR was still not clear. Thirdly, there were only 186 cases in their study; the sample was small. Thus, the prognostic value of AGR in GC was worthy of further study.

In our study, we included 862 cases with and without radical surgical, and the pretreatment AGR was an independent prognostic factor for patients with GC in multivariate analysis in both groups. Furthermore, after stratification by TNM category, preoperative AGR remained significantly prognostic, especially in advanced TNM category patients. Previous studies had reported that chronic inflammation was strongly correlated with the invasion and metastasis of GC [20, 21]. The tumor microenvironment was constituted by inflammatory cells, inflammatory cytokines, and inflammatory chemokines [22]. The formation of tumor microenvironment could lead to the infiltration of immunosuppressive cells such as myeloid derived suppressor cell, regulatory T cell and tumor associated macrophage, upregulation of a series of proinflammatory factors, and accumulation of a large number of inflammatory cytokines such as IL-6, IL-11, TGF-*β*, and MMP. These cytokines could promote the immune escape and metastasis of tumors by STAT3, NF-*κ*B signal pathway, and so on [23–27].

As clinical routine detection indexes, ALB and GLB were the main proteins in human body. ALB played an important role in material transport and the maintenance of plasma osmotic pressure. It not only reflected the nutritional status of the body, but also represented the level of inflammation [11]. Cytokines, such as IL-6 and TNF, were produced by the inflammatory response, which could inhibit the synthesis of ALB in liver cells [28]. A long term chronic inflammation could lead to vascular endothelial damage and increase vascular permeability, which could increase the level of ALB in interstitial fluid and result in decreasing of serum ALB. Previous studies had confirmed that a low level of ALB was associated with poor prognosis in a variety of malignant tumors, including GC [29], colorectal cancer [30], lung cancer [31], and breast cancer [32]. GLB was produced by immune organs and reflected the immune state [33], which contained a lot of acute reactive proteins such as *α*_1_-antitrypsin, *α*_2_-macroglobulin, and haptoglobin. In the stimulation of inflammation, the level of serum GLB increased rapidly by several inflammatory indicators; it was also correlated with poor prognosis in several malignant tumors [34, 35].

Although previous studies demonstrated ALB and GLB to be significant prognostic factors, which would be influenced by many factors, body dehydration or fluid retention had great impacts on ALB and GLB levels; however they had little influence on AGR. Previous studies had shown that a decreased pretreatment AGR level predicted poor survival in several malignant tumors [14–17]. In our study, AGR was identified to be an independent prognostic factor for GC after adjusting by confounding prognostic factors in both the testing group and the validation group. These findings proved that pretreatment AGR was a promising inflammatory biomarker that would improve the prognostic value for patients with GC.

In this study, we found that AGR had significant prognostic value in patients with GC. But there were still some limitations in our study. Firstly, prospective studies were needed to confirm our conclusions because it was a retrospective analysis. Secondly, there was no uniform cutoff value for AGR so far, so different statistical methods may obtain different cutoff value, while the method we used was based on the R language, which had been proved by other studies with high reliability.

The present data show that preoperative AGR is strongly associated with overall survival in GC. Both ALB and GLB are convenient low-cost indicators that are routinely measured in clinical practice. Thus, the AGR is highly feasible and is validated in our study. In conclusion, our study demonstrated that AGR is an independent biomarker of poor survival and could be employed as a prognostic tool in patients with GC.

## Supplementary Material

Prognostic value of AGR for overall survival in whole GC patients by univariate and multivariate analyses.

## Figures and Tables

**Table 1:** The clinicopathological characteristics of gastric cancer patients.

Characteristic	The testing group	The validation group
	Median (25th–75th percentile) or number (%)	Median (25th–75th percentile) or number (%)
Gender		
Male	288 (66.8)	298 (69.1)
Female	143 (33.2)	133 (30.9)
Age (years)		
≤59	222 (51.5)	232 (53.8)
>59	209 (48.5)	199 (46.2)
TNM category (AJCC, 7th)		
I	66 (15.3)	58 (13.5)
II	110 (25.5)	100 (23.2)
III	201 (46.6)	208 (48.3)
IV	54 (12.5)	65 (15.1)
N stage (AJCC, 7th)		
N0	130 (30.2)	141 (32.7)
N1	301 (69.8)	290 (67.3)
T stage (AJCC, 7th)		
T1	54 (12.5)	40 (9.3)
T2	46 (10.7)	61 (14.2)
T3	142 (32.9)	138 (32.0)
T4	189 (43.9)	192 (44.5)
M stage (AJCC, 7th)		
M0	366 (84.9)	349 (81.0)
M1	65 (15.1)	82 (19.0)
Primary tumor size (cm)		
<4.0	178 (41.3)	171 (39.7)
≥4.0	253 (58.7)	260 (60.3)
Tumor location		
Proximal	218 (50.6)	239 (55.5)
Remote	160 (37.1)	147 (34.1)
Other	53 (12.3)	45 (10.4)
Degree of differentiation		
Poorly or not differentiated	253 (58.7)	250 (58.0)
Moderately differentiated	174 (40.4)	179 (41.5)
Well differentiated	4 (0.9)	2 (0.5)
Tests		
CRP	1.78 (0.12–155.32)	1.91 (0.12–278.56)
ALB	41.6 (25.1–49.1)	41.9 (27.50–51.00)
GLB	27.3 (12.8–41.8)	27.45 (16.90–42.00)
PLT	240.90 (88.00–869.00)	235.00 (77.00–641.60)
Neutrophil	3.7 (0.7–17.1)	3.80 (1.20–19.37)
Lymphocyte	1.6 (0.2–3.7)	1.70 (0.23–3.80)

**Table 2:** Prognostic value of AGR for overall survival in the testing group of GC patients by univariate and multivariate analyses.

Characteristics	Univariate analysis			Multivariate analysis		
	Hazard ratio	95% CI	*p* value	Hazard ratio	95% CI	*p* value
Sex						
Male versus female	0.886	0.603–1.302	0.538			
Age (yr)						
≤59 versus >59	1.313	0.922–1.870	0.131			
Primary tumor size (cm)						
<4.0 versus ≥4.0	1.855	1.182–2.909	0.007	1.451	0.828–2.544	0.193
Tumor location						
Proximal versus remote versus other	1.366	1.046–1.784	0.022	1.224	0.864–1.735	0.255
Degree of differentiation						
Poorly versus moderately versus well	1.080	0.560–2.083	0.818			
Distant metastasis						
No versus yes	6.989	4.776–10.228	<0.001	2.361	1.216–4.585	0.011
Surgery						
No versus yes	0.509	0.337–0.768	<0.001	1.467	0.745–2.887	0.268
TNM category (AJCC, 7th)						
I + II versus III + IV	4.577	2.859–7.326	<0.001			
NLR						
≤2.08 versus >2.08	1.959	1.344–2.855	<0.001	1.269	0.748–2.153	0.378
PLR						
≤140.63 versus >140.63	1.923	1.330–2.778	0.001	1.106	0.628–1.950	0.727
AGR						
≤1.50 versus >1.50	0.626	0.438–0.859	0.010	0.584	0.351–0.973	0.039

**Table 3:** Prognostic value of AGR for overall survival in the validation group of GC patients by univariate and multivariate analyses.

Characteristics	Univariate analysis			Multivariate analysis		
	Hazard ratio	95% CI	*p* value	Hazard ratio	95% CI	*p* value
Sex						
Male versus female	1.121	0.81–1.552	0.49			
Age (yr)						
≤59 versus >59	1.015	1.002–1.029	0.021	1.080	0.705–1.653	0.723
Primary tumor size (cm)						
<4.0 versus ≥4.0	1.713	1.180–2.487	0.005	1.319	0.800–2.174	0.278
Tumor location						
Proximal versus remote versus other	1.096	0.876–1.371	0.423			
Degree of differentiation						
Poorly versus moderately versus well	1.561	0.912–2.672	0.104			
Distant metastasis						
No versus yes	5.375	3.898–7.410	<0.001	2.939	1.781–4.850	<0.001
Surgery						
No versus yes	0.430	0.307–0.626	<0.001	0.973	0.585–1.620	0.917
TNM category (AJCC, 7th)						
I + II versus III + IV	6.205	3.035–12.678	<0.001			
NLR						
≤2.08 versus >2.08	1.946	1.415–2.676	<0.001	1.766	1.136–2.745	0.011
PLR						
≤140.63 versus >140.63	1.630	1.199–2.215	0.002	1.373	0.885–2.129	0.157
AGR						
≤1.50 versus >1.50	0.649	0.478–0.882	0.006	0.578	0.373–0.897	0.015

## Abbreviations

GC: Gastric cancer
ALB: Albumin
GLB: Globulin
AGR: ALB/GLB ratio
OS: Overall survival.
