# Supplementary material for: Clinical Significance of Preoperative Albumin and Globulin Ratio in Patients with Gastric Cancer Undergoing Treatment
Source: Biomed Res Int. 2017 Mar 23;2017:3083267. doi: 10.1155/2017/3083267 (PMC5382292; doi:10.1155/2017/3083267)
Supplement: Supplementary file 1 — Prognostic value of AGR for overall survival in whole GC patients by univariate and multivariate analyses. [file 3083267.f1.pdf]

Supplement 1. Prognostic value of AGR for overall survival in whole GC patients by univariate and multivariate analyses.

characteristics	Univariate analysis			Multivariate analysis		
	Hazard ratio	95% CI	*p* value	Hazard ratio	95% CI	*p* value
Sex						
Male vs. Female	0.998	0.778-1.279	0..985			
Age (yr)						
≤ 59 vs. > 59	1.350	1.073-1.698	0.010	1.266	0.913-1.755	0.157
Primary tumor size (cm)						
< 4.0 vs. ≥4.0	1.779	1.335-2.370	<0.001	1.188	0.817-1.727	0.366
Tumor location						
Proximal vs. Remote vs. Other	1.195	1.007-1.419	0.042	1.247	0.997-1.561	0.054
Degree of differentiation						
Poorly vs. Moderately vs. Well	1.336	0.882-2.023	0.171			
Distant metastasis						
No vs. Yes	6.113	4.787-7.807	<0.001	2.247	1.504-3.356	<0.001
Surgery						
No vs. Yes	0.469	0.358-0.613	<0.001	1.229	0.819-1.843	0.320
TNM category (AJCC, 7th)						
I+II vs. III+IV	5.320	3.246-8.721	<0.001	2.962	1.627-5.393	<0.001
ALB						
≤41.7 vs. >41.7	0.695	0.552-0.875	0.002	0.963	0.690-1.344	0.825
GLB						
≤27.4 vs. >27.4	1.205	0.957-1.517	0.112			
NLR						
≤2.08 vs. > 2.08	1.934	1.517-2.466	<0.001	1.442	1.028-2.023	0.034
PLR						
≤140.63 vs. >140.63	1.730	1.367-2.190	<0.001	1.023	0.721-1.453	0.897
AGR						
≤1.50 vs. > 1.50	0.648	0.514-0.817	<0.001	0.630	0.451-0.879	0.007

In univariate analysis, age (HR:1.50, 95%CI=1.073-1.698, *p*=0.010), primary tumor size (HR:1.779, 95%CI=1.335-2.370, *p*<0.001), tumor location(HR:1.195, 95%CI=1.007-1.419, *p*=0.042), distant metastasis (HR:6.113, 95%CI=4.787-7.807, *p*<0.001), surgery (HR:0.469, 95%CI=0.358-0.613, *p*<0.001), the TNM category (HR:5.320, 95%CI=3.246-8.721, *p*<0.001), ALB (HR:0.695, 95%CI=0.552-0.875, *p*=0.002), PLR (HR:1.730, 95%CI=1.367-2.190, *p*<0.001), NLR (HR:1.94, 95%CI=1.517-2.466, *p*<0.001) and AGR (HR:0.648, 95%CI=0.514-0.817,

p<0.001) were significantly associated with OS. Multivariate analysis (Cox proportional hazards model) identified distant metastasis (HR: 2.247, 95%CI=1.504-3.356, *p*<0.001), the TNM category (HR:2.962, 95%CI=1.627-5.393, *p*<0.001), NLR (HR: 1.442, 95%CI=1.028-2.023, *p*=0.011) and AGR (HR: 0.630, 95%CI=0.451-0.879, *p*=0.007) as independent prognostic factors. ALB and GLB were not significantly associated with OS.
